# Transitioning the Adolescent with IBD from Pediatric to Adult Care: A Review of the Literature

**DOI:** 10.1155/2015/853530

**Published:** 2015-05-04

**Authors:** Natasha Bollegala, Geoffrey C. Nguyen

**Affiliations:** Mount Sinai Hospital, Joseph and Wolf Lebovic Health Complex, 437-600 University Avenue, Toronto, ON, Canada M5G 1X5

## Abstract

The incidence of inflammatory bowel disease (IBD), comprising Crohn's disease (CD) and ulcerative colitis (UC), has increased in pediatric populations over the last decade. Patients diagnosed during childhood often survive well into adulthood, and therefore their healthcare requires transfer to an adult gastroenterologist, usually at age 18 years. Transition has been defined in the literature as the “purposeful planned movement of adolescents and young adults with chronic conditions from child-centered to adult-oriented health care systems” (Blum et al., 1993). The purpose of this review is to establish the current state of knowledge regarding the transition from pediatric to adult care in IBD. This review highlights that developmentally appropriate transitional care is now recognized as a healthcare priority and thoughtful targeted intervention is needed.

## 1. Introduction

Approximately 30% of Crohn's disease (CD) and 20% of ulcerative colitis (UC) present before the age of 20 [1, 2]. In a US population-based study, the incidence of pediatric IBD was 7.05/100,000 (with a twofold predominance in CD) and appears to be rising [3, 4]. Based on this, the projected prevalence of pediatric onset IBD in the US alone is between 45,000 and 100,000 with about 10,000 new cases annually [2].

Children diagnosed with IBD eventually require transfer of care to an adult gastroenterologist. The transition to adult care presents a challenge because of inherent differences between healthcare models. Pediatric healthcare is family focused, multidisciplinary, and reliant on parental involvement for consent and guidance. In contrast, adult gastroenterology is focused on the individual patient; is carried out by single providers; and advocates patient independence [5]. The transition period occurs as children are potentially graduating from secondary education and entering into postsecondary education or a vocation. They are becoming more financially independent and may be leaving the family home. With these overlapping events, there is concern that patients with PO-IBD are at risk for adverse clinical outcomes. The potential for this arises from new and unfamiliar relationships with adult healthcare providers, diminished parental oversight, noncompliance related to the developmental profile of adolescence, economic barriers limiting access to pharmacological therapy, and an underdeveloped understanding of the disease.

Transition-related healthcare interventions can be characterized as the “purposeful planned movement of adolescents and young adults with chronic physical and medical conditions from child-centered to adult-oriented health care systems” [6]. Escher clearly identified 3 goals in the process of transition: “(1) to get the patient ready for transfer, having attained specific skills and knowledge; (2) to get the parents ready for transfer; (3) to get the adult gastroenterologist ready and well informed at the time of transfer” [7]. The delivery of transitional care, although debatable in terms of format, should be in accordance with a patient's age and mental development. It should focus on honing skills in communication and decision-making that will enable young adults to actively engage in their IBD care. Successful transition programs should characteristically promote continuity of care, improve treatment adherence and disease knowledge, encourage independent disease management, and build confidence in the new adult healthcare team, all with the overarching goal of improving or maintaining disease control [6].

Transition has been identified as a health services priority area [8]. Despite this, there have been few evidence based methods developed to clinically and cost effectively transition teens with IBD to adult care. We have conducted this review of the literature to identify current recommendations, explore the current state of transitional care, and present available evidence based literature for clinicians faced with this increasingly common scenario.

## 2. IBD Transition Recommendations

The North American Society for Pediatric Gastroenterology, Hepatology, and Nutrition (NASPGHAN) has published a set of recommendations pertaining directly to the transition of patients with childhood onset IBD from pediatric to adult care [5, 9, 10]. Although based mostly on expert pediatric consensus, these recommendations contain practical tips and tools.

The NASPGHAN recommendations outline the following 5 steps for providing transitional care:

1. Start seeing the patient without their parents to build independence and self-reliance.
2. Discuss the benefits of transition to an internal medicine gastroenterology practice. For example, pregnancy and fertility issues, ongoing cancer surveillance, and other adult health problems may be more adequately addressed by an adult provider.
3. Select an adult gastroenterologist who is interested in the unique needs of young adults with childhood onset of disease. Some patients may prefer to meet the anticipated adult provider in advance of complete transfer of care. They may also request a final visit with the pediatric provider that postdates the first adult care visit.
4. Provide all necessary medical records and summaries to the adult healthcare provider and the patient.
5. Allow flexibility in transition timing. The timing of transition requires a balance between the patient's maturity and readiness for transfer of care. Regardless, transfer of care in most North American institutions is mandatory by age of 18 years. Therefore, early efforts at achieving adequate transition preparedness are important.

Another practical recommendation pertains to the documents or information patients should bring with them to the first adult appointment with their adult gastroenterologist. These suggestions are similarly echoed by Hait et al. [5].

1. A medical summary: This should, at minimum, include the date of diagnosis, location and severity of disease, surgeries and complications, medical therapies used (including dose and duration), and adverse reactions to medications. Although not included in this set of recommendations, a complete vaccination history and results of TB testing (if done) may also be included.
2. Information regarding current drug plan coverage: Although not included specifically in these recommendations, plans regarding the ultimate termination of parent provided drug insurance and plans for long-term continuing coverage are important. This is particularly critical if the patient requires anti-TNF therapy and may require early liaising with government-based extended health insurance and representatives from the appropriate pharmaceutical companies.
3. A calendar or schedule for booking future appointments and tests: Although not included specifically, given the widespread use of smart phones and other handheld electronic devices, making use of these is a practical method for recording health information.
4. A list: A list of important names and relevant contact information of the patient's primary care provider, local pharmacy, other involved healthcare providers, and transportation company (if applicable) should be provided.

Lastly, NASPGHAN has produced age and developmentally appropriate checklists for patients and pediatric healthcare providers to ensure that the skills necessary for successful transition are introduced incrementally as achievable stepwise goals throughout adolescence. The transition model suggested begins at 12 years, allowing ample opportunity to anticipate and practice the new roles for family and patient. Adolescent patients learn to problem solve and gain a sense of mastery, thereby increasing their sense of empowerment, self-efficacy, and self-determination.

Leung et al. provide recommendations specifically for adult gastroenterologists which again are based on expert opinion [11]:

1. Be aware that adolescent patients entering the adult healthcare system may not be prepared or knowledgeable about the differences between pediatric and adult care.
2. Collaborate with the pediatric gastroenterologist prior to transfer of care. This may involve developing a transition plan which integrates the accepting provider, transition-related tasks that must be mastered prior to transfer of care, and a plan regarding continuing health insurance.
3. Anticipate questions from the patient. Be aware of educational material available and resources.
4. Educate the patient regarding the adult healthcare system.
5. Realize that the first few appointments with a transitioning patient may be longer.
6. Be prepared for ongoing parental involvement and the fact that patient independence is gradual.

## 3. IBD Transition Models

There has been little documentation regarding the development and evaluation of transition models and their associated clinical and cost effectiveness in the IBD population. Escher describes three possible structured transition options, although these have not been individually studied: (1) yearly combined visits with the pediatric and adult gastroenterologist starting at age 14 years; (2) three alternating visits with a pediatric and adult gastroenterologist in the year prior to transfer of care; (3) one combined final visit with the pediatric and adult gastroenterologist, occurring at the time of transfer [7].

NASPGHAN supports the importance of collaborative transition planning and the potential for a joint visit to facilitate communication between pediatric and adult healthcare providers as well as with patients and parents. This venue allows concrete planning around current and future treatment [9].

Davies and Jenkins, based on expert opinion, suggested three principles that should guide the development of a transition program for adolescent patients with chronic gastrointestinal disease. (1) Choose a time that is convenient for the patient. (2) Both pediatric and adult gastroenterologists should be present in addition to other healthcare providers (e.g., specialist nurses). (3) A medical summary should be provided to healthcare practitioners as well as the patient and their family in advance of the transition appointment. They also recommended that the focus of the transition appointment should be to review the patient's medical management [12].

Dabadie and colleagues have been the only authors thus far to critically evaluate the transition process and specifically the role of a joint transition visit, at a single French center [13]. Patients were invited to attend 1 joint visit with pediatric and adult care providers. Questionnaires were mailed to patients and parents regarding their retrospective perceptions of transitional care after at least 1 year of adult follow-up. Forty-eight patients underwent transition during the study period. The majority (71%) transferred to an adult gastroenterologist within the same hospital. A less severe disease course was associated with patients who transferred their care to a practitioner outside of the university center. Among those who stayed at the university hospital, 79% attended the joint visit. Patients who had active disease or perianal CD were more likely to attend. All patients deemed the joint visit to be beneficial for transmitting information, and the vast majority (93%) felt that it facilitated building confidence in the new provider. A recently completed clinical trial has looked at the effectiveness of two pediatric-adult joint clinics in improving patient satisfaction and reducing IBD flares and results from this study are forthcoming [14].

Although the most effective format of transitional programming is yet to be determined, it seems clear that all models identify the communication and confidence building as key priorities. Other potential components of a pediatric-adult transition model include educational forums, alternating visits between pediatric and adult clinic sites, and the integration of a dedicated nurse transition coordinator [11].

## 4. Current Status of Transitional Care

There is a relative dearth of literature addressing the current status of transitional care. Most of the available data on transitional services comes from the United Kingdom (UK). The 2008 UK IBD Audit 2nd Round identified the standard in IBD transition-related support services as “regular (usually 1 or 2 per year) transition clinics involving pediatricians and adult gastroenterologists for handover of patients to adult services.” In 2006, only 23% (42/180) of sites provided pediatric to adult handover clinics, and this remained unchanged in 2008 with only 25% (54/205) of sites participating. The UK IBD Audit 3rd Round indicated that 39% of the 202 adult sites administered care for IBD patients aged 16 years and under. Of these, 73% indicated that care was provided in conjunction with a pediatric gastroenterologist or a pediatrician with an interest in gastroenterology. Less than half of these sites (46%) reported that they had a specific pediatric to adult transition policy. Among the adult IBD centers that provided care to patients aged 16 years and under, the proportion of various multidisciplinary healthcare providers with pediatric experience was highly variable: surgeons (47%), radiologists (58%), dietitians (72%), and IBD/gastroenterology nurse specialists (31%) [15, 16].

A survey of pediatric gastroenterologists and hepatologists in the UK and Ireland revealed that 12 of 15 clinics which responded had a transition program in place (6 took place in the pediatric center and 3 in the adult center and 3 alternated between the pediatric and adult centers). The average age of transitioning patients was 16 years and transition clinics ran from 3 to 6 times per year. The median number of transition clinic visits prior to transfer to adult care was 3. There was also variability on how these clinics were administered with 9 out of 12 sites employing a nurse specialist [17].

Unfortunately, there has been no documentation regarding the current state of transitional services in North America.

## 5. Key Stakeholders

Facilitating the transition from pediatric to adult care requires an appreciation for the perspectives and concerns of each of the key stakeholders. Paine et al. performed semistructured interviews on 12 IBD healthcare providers (both pediatric and adult) to determine measurable outcomes in transitional care, barriers to transitional care, and facilitators. Emergent themes explored in detail the role of each stakeholder. The following considerations are based primarily on expert opinion, including those of the Paine group and this review's authors.

### 5.1. Patients

The experience of childhood onset disease can have a profound impact on the cognitive and emotional state of an adolescent with IBD. These teenagers must struggle to overcome a habitual reliance on their caregivers and health providers, which was necessary earlier in the course of their disease, in order to achieve stepwise independence as they transition to adult care. These adolescents may also experience uncertainty with regard to future wellness, and the impact of chronic disease on educational achievement, employment prospects, and peer relationships. These concerns may also elicit a reluctance for graduated independence from parents.

The ongoing cognitive development of adolescents with IBD is an important educational consideration. IBD-related education must be repeatedly reinforced, and providing concrete examples is more effective in these young adults. Creating an environment in which adolescents feel comfortable posing questions and revealing their knowledge gaps is similarly important [9].

Developmental maturity has been identified as a key component to transition success with the ability to abstract reason critical for problem solving and disease management. Psychosocial difficulties, communication challenges with healthcare providers, and motivational problems may similarly present barriers [18].

### 5.2. Parents

The focus of adult care is on the patient, with an associated expectation of independence, and this may leave family members (specifically parents) feeling neglected. Parents may feel uncomfortable with the new expectation that they trust their increasingly independent adolescent and a new unfamiliar care team with the details of their child's healthcare [18]. They also may experience a profound sense of loss when forced to leave behind their pediatric team who were a source of support and guidance [19–23]. Parents need to be encouraged to have confidence in their child's expanding self-management skills and be reminded that any interventions taken to build independence must be supported and continued at home [9].

### 5.3. Pediatric Providers

The pediatric gastroenterologist has likely developed a relationship with the patient and family that is based on experience and earned trust. The ensuing sense of attachment may lead to reluctance to transfer care to a practitioner whom they feel may lack expertise to deal with childhood onset IBD. Thus, early open communication with the receiving adult gastroenterologist to ensure complete transfer of medical information is crucial for establishing a functional and supportive relationship between providers [18].

### 5.4. Adult Providers

Adult gastroenterologists, often working within a single-provider system, may not have access to the multidisciplinary environment to which pediatric IBD patients have become accustomed. These providers may additionally lack the confidence and/or training to treat adolescents with childhood onset IBD [18]. They may perceive the patient as immature and the family too involved or demanding. Recently transitioned IBD patients may require more time in the office. Adult gastroenterologists must be ready and willing to invest more time into caring for these complex patients and be prepared to address the expectations and fears of patients and family members.

### 5.5. Health Systems

Multiple barriers exist when transferring a patient from the pediatric to adult healthcare system. It can be difficult to access medical records from previous providers and institutions. Securing health insurance may be a significant issue as adolescents may be removed from their parents' insurance within a few years of transition. This transition of insurance may pose challenges for maintaining current drug regimens, particularly biologics. Young adults (18–24 years) have among the highest rates of being uninsured. Over a quarter of young adults with disabling, chronic health condition have no health insurance; more than a third of these individuals have reported unmet health needs as a result [24, 25]. Even in countries with universal healthcare, such as Canada, prescription medication for most citizens is not covered by the government and requires private drug coverage.

## 6. IBD Transition: A Review of the Evidence

### 6.1. Knowledge of Medication

Fishman and colleagues assessed the ability of 454 American patients with IBD aged 10 years and older to recall medication name, dose, and adverse effects. Most patients were successful at recalling their IBD medications, though nearly 10% of patients on a biologic failed to list it. They were less proficient at enumerating dosages, being successful 50–75% of the time. Only 22–32% of pediatric IBD patients were aware of adverse effects of their medications, which did improve with age [26]. Another survey showed that almost all patients were aware of their type of IBD and were comfortable describing their disease to a friend. While most relied on their parents to manage the logistics of arranging clinic appointments and medication pick-up, they did assume responsibility for doing most of the talking during the clinic visit [27].

### 6.2. Medication Nonadherence

Adherence among children with chronic disease is reported to be 50% and is typically lowest during disease remission. A study of 74 adolescents with IBD reported that the most common barriers to oral medications were “lack of time,” “forgetting,” “being away from home,” and “interference with an activity” [28]. Reasons for discontinuing medications included the presence of side effects, feeling well, and believing that the medications were not working. The use of monotherapy and less frequent dose administration were both associated with fewer barriers to adherence [29, 30].

### 6.3. Inaccurate Assessments of Transition Readiness

There appears to be poor correlation between clinicians' perception of transition-related literacy readiness and interactive/functional health literacy measures. Factors predictive of literacy readiness for transition include age, white race, and insurance status. Only 16% of adolescents with IBD have been deemed to have adequate transition readiness [31].

### 6.4. Healthcare Provider Perceptions of Transition Requirements

Pediatric gastroenterologists are more likely than adult gastroenterologists to stress the importance of structured individualized transition (79% versus 47%, *p* = 0.001). Pediatric gastroenterologists also believe in the importance of being in clinical remission at the time of transfer to adult care. Adult gastroenterologists cite deficiencies in patient knowledge of the disease and its treatment as well as coordination of care during the transition period. Commonly cited barriers to providing transitional care include lack of space and time, lack of funding, and lack of supportive services. Pediatric gastroenterologists, on the other hand, identify insufficient trust in adult clinical services and lack of interest by adult colleagues as barriers to successful transition. Both pediatric and adult gastroenterologists identify suboptimal training in adolescent medicine among adult providers, with only 73% feeling competent in this area [5, 32]. A survey of adult gastroenterologists described their expectations of transitioning patients to include knowledge about medications, prior medical history, and the impact of smoking and drugs on health. They expected to receive accurate information from the pediatric gastroenterologist at the time of transfer.

### 6.5. Evidence Based Transition Tools

#### 6.5.1. Personal Medical History

The MyHealth Passport is a customized, wallet-sized card providing patients, families, and caregivers with a comprehensive summary of the patient's medical information. It is a free application available through the SickKids Hospital website and has been studied in a cross-sectional format showing that patients and their parents are equally likely to answer questions correctly with respect to disease characteristics and treatment when compared to medical charts. Parents, however, are more accurate when recalling health services resources (e.g., insurance provider and pharmacy details) [33].

#### 6.5.2. IBD Education

Boamah et al. developed a theory-based educational program for American adolescents with IBD designed to improve IBD-specific knowledge using an interactive multimedia CD-ROM that is web-based and available free of charge at https://dl.dropboxusercontent.com/u/182888348/_IBD_CD/_IBDedu.swf. The program includes components on what IBD is, symptoms and diagnosis, complications, medications, nutrition, social functioning, and personal testimonials. After 30 minutes of self-directed exploration of the curriculum, individuals demonstrated improved knowledge scores, as measured by Crohn's and Colitis Knowledge (CCKNOW) questionnaire, immediately after the intervention and up to 9 months afterward. Patients had increased their knowledge of anatomy, gastrointestinal function, general IBD knowledge, complications, and treatment. Importantly, participants remarked that they were more likely to use the educational program at an ambulatory visit rather than at home or school because of lack of motivation [34].

#### 6.5.3. Self-Efficacy Assessment

Zijlstra and colleagues have developed and validated an IBD-specific self-efficacy questionnaire for patients and their parents that queries the following domains: general independence, knowledge of IBD, diagnostic tests, and treatment, self-efficacy in medication use, actual behavior regarding medication use in the past week, skills for independent transition clinic visits, actual behavior during transition clinic visits, coping with IBD, knowledge of the transition process, and readiness for transfer to the adult gastroenterologist. Median self-efficacy scores of adolescents with IBD varied from 70 to 100%. Parents tended to overestimate their child's self-efficacy relative to how the child scored themselves especially with respect to knowledge of IBD and diagnostic tests, self-management of medication use and transfer readiness. Male gender, higher education level, and length of time since first transition clinic were positive predictors of self-efficacy [35].

## 7. Other Chronic Disease Transition Populations

A number of other childhood onset chronic diseases exist and transition-related literature is at varying stages of development. Amongst the best developed are congenital heart disease (CHD), cystic fibrosis (CF), and type 1 diabetes mellitus (T1DM). Other disease states with earlier development of transition knowledge include childhood onset obesity, HIV, palliative care, oncology, liver transplantation, eating disorders, and other endocrine disorders such as congenital adrenal hyperplasia. Regardless, the majority of research in this area has been descriptive and reports qualitative outcomes. Many of the recommendations are based on expert opinion and suggested that transition practices are not evidence based. There have been attempts to change this. IBD transition literature comparatively is still small in scope and while it has successfully created clinical targets, identified deficiencies in knowledge, adherence, and transition readiness assessments, and found differential expectations amongst the pediatric and adult provider communities, there are still few evidence based tools to assist in the transition process and a relative absence of transition models that have been critically appraised. Ultimately we aim to improve the transition experience and ameliorate objective health outcomes such as compliance, loss-to-follow-up, lapses in care, knowledge, disease activity, and emergent health resource utilization. We have not yet shown that an improvement in transitional care will achieve this.

## 8. Conclusion

This review highlights that age and developmentally appropriate transitional care are recognized by healthcare providers as a healthcare priority. It is also evident that there has been relatively little literature in this area to develop and evaluate transition models and transition tools and to determine their cost and clinical effectiveness. The majority of our knowledge in this area pertains to joint pediatric-adult visits, medication adherence, patient and parent knowledge and patient, parent and healthcare provider perceptions.

Transitional efforts ultimately aim to improve patient, parent, and healthcare provider satisfaction while improving disease activity and control. In this young population destined to experience decades of disease and exposure to therapeutics, we aim to reduce dependence on steroids and improve participation and achievement in educational and occupational endeavors as well as optimize psychosocial wellbeing.

For hospitals and healthcare providers caring for adolescents and their families during this vulnerable period, what is lacking is an evidence based approach. It remains uncertain which models for transitional care are effective and which are most appropriate for childhood onset IBD. Future research needs to address whether the model of care should vary depending on the severity of the underlying IBD and whether clinical outcomes actually improve as a result of structured transition care. Importantly, much of the literature in this area is driven by pediatric gastroenterologists without commensurate partnership from adult practitioners. Provision of opportunities for education and training in adolescent medicine may stoke interest, instill confidence, and garner greater participation in the pediatric-adult transition process amongst adult gastroenterologists.
